# Acceptance of E-Learning Devices by Dental Students

**DOI:** 10.2196/med20.2767

**Published:** 2013-08-14

**Authors:** Peter Schulz, Keyvan Sagheb, Harald Affeldt, Hannah Klumpp, Kathy Taylor, Christian Walter, Bilal Al-Nawas

**Affiliations:** ^1^Department of Oral and Maxillofacial SurgeryUniversity Medical CenterJohannes Gutenberg UniversityMainzGermany; ^2^Ressort Research and TeachingUniversity Medical CenterJohannes Gutenberg UniversityMainzGermany; ^3^Institute for Medical Biometry, Epidemiology and InformaticUniversity Medical CenterJohannes Gutenberg UniversityMainzGermany

**Keywords:** e-learning activity, computer, tablet PC, smartphone, Internet

## Abstract

**Background:**

E-Learning programs and their corresponding devices are increasingly employed to educate dental students during their clinical training.

**Objective:**

Recent progress made in the development of e-learning software as well as in hardware (computers, tablet PCs, smartphones) caused us to more closely investigate into the habits of dental students in dealing with these learning techniques.

**Methods:**

Dental students during their clinical training attended a survey compiled in cooperation with biostatisticians. The questionnaire probands were asked to complete based on previous surveys of similar subjects, allowing single as well as multiple answers. The data, which were obtained with respect to the learning devices students commonly employ, were compared with their internet learning activities.

**Results:**

The e-learning devices utilized are of heterogeneous brands. Each student has access to at least one hardware type suitable for e-learning. All students held mobile devices, about 90 percent employed laptops, and about 60 percent possess smartphones. Unexceptional all participants of the survey acknowledged an unlimited internet access. In contrast, only 16 percent of students utilized tablet PCs. A detailed analysis of the survey outcome reveals that an increasing use of mobile devices (tablet PC, smartphone) facilitates internet learning activities while at the same time utilization of computers (desktop, laptop) declines.

**Conclusions:**

Dental students overwhelmingly accept e-learning during their clinical training. Students report outstanding preconditions to conduct e-learning as both their access to hardware and to the internet is excellent. Less satisfying is the outcome of our survey regarding the utilization of e-learning programs. Depending of the hardware employed only one-third to barely one-half of students comprise learning programs.

## Introduction

The use of electronic devices in dental medicine for patient care, teaching, and learning, respectively, has been widely accepted [1-3]. As consequence of this development it became evident in recent years that internet-based learning increased its attraction for students at large [4,5], including those of dental medicine [6,7]. However, utilization of these new media depends on several criteria, specifically on the availabilty of a convenient hard and software as well as access to a high speed internet [8]. In this context authors repeatedly refer to the Web 2.0 as a basic social software for a successful implementation of e-learning [9].

The new generation of mobile phones and tablet personal computers (PCs) appears to perfectly adapt to the general framework of e-learning techniques presently available [10]. Taking account of these facts this investigation pursues two major issues. First, we examined current e-learning activities of dental students with reference to their use of specific e-learning devices that are desktop computers, tablet PCs (iPads), and smartphones, respectively. Second, probands were also questioned regarding a preferred utilization of commonly available teaching programs, which can possibly be linked to special e-learning devices.

## Methods

Dental students (n=141) in their second and third clinical semester of the University Mainz were asked to attend an optional survey, nobody refused. No student was excluded since all of them met essential criteria such as mastering the German language and complying with basic technological literacy regarding the electronic devices here under discussion. Probands were encouraged to request assistance in case they believe the questionnaire implies ambiguous issues. The participants were invited to independently complete a machine-readable form within 20 minutes. This procedure appears superior over online surveys as an increased reliability of results may be expected [11].

The queries put emphasis on different domains of learning preferences as well as on hardware devices individual students had access to. The inquiry schedule also considers previously conducted surveys regarding the percentage of internet-based learning [6], and allows single as well as multiple answers. Table 1 displays an outline of the questionnaire the probands were confronted with.

Further information was requested concerning the operation systems (OS) the devices are equiped with (Table 1, question 4), as 2 OSs are mainly installed on hardware devices. That is the OS introduced by Apple (Apple Inc., Cupertino, CA, USA) for iPhones or iPads (iOS), and the Android OS provided by Google (Google Inc., Mountain View, CA, USA). The market share for the year 2012 in Germany of both OSs reveals a 77% rate for iOS and 19% for Android OS [12]. Evaluation of question 2, considers the quantity of students in relation to the hardware (eg, tablet PCs, smartphones, laptops) each participant utilizes. The obtained data were correlated with the internet-based learning activities of the probands.

The questionnaire was compiled in cooperation with biostatisticians of the Center for Quality-Management and Development, Johannes Gutenberg University Mainz. These experts also conducted the machine read-out and processing of the completed questionnaires, employing the statistical analysis programme SPSS 16.0 (Statistical Package Social Sciences; IBM Inc., Chicago. IL, USA). The survey analysis did not differentiate between male and female participants as well as of their degree of clinical education.

**Table 1:** Outline of the questionaire provided to students.

Question #	Question
1	Do you have an Internet access from your home? If „yes“, which kind?
2	Define the percentage of your internet-based learning activity.
3	Which type of computer (desktop, notebook, netbook) are you utilizing?
4	Which kind of mobile device (tablet PC, smartphone) and operation system (OS) are you working with?
5	Are you mainly utilizing the university’s e-learning offers at home or where else?
6	Which medium (iOS apps, Android apps, computer programs) are you deploying for learning activities?

## Results

The study comprises 141 students during their clinical education, 42 males and 99 females. The gender distribution roughly matches the notification (10% variance) officially provided [13]. Preliminary talks unveiled that each student participating in the survey employed any e-learning device and owned at least one device useful for e-learning activities. Analysis of the survey confirms this statement, and details that an overwhelming number of probands (125/141, 89%) utilized laptops for e-learning activities. Beyond that, all students reported an unlimited Internet access. Furthermore, all students hold mobile devices, although tablet PCs and smartphones employed for e-learning can be assigned to only 75% (106/141) of probands. Apparently, less than two-thirds (83./141, 59%) of students questioned own smartphones, and a minority (23/141, 16%) has tablet PCs at one's disposal. Examining the devices with respect to their OS it appears that iOSs prevail. The overall conclusion drawn from this data clearly demonstrates that at least with respect to the hardware (e-devices) clinical dental students are excellently equipped to accomplish e-learning requirements.

**Figure 1:**
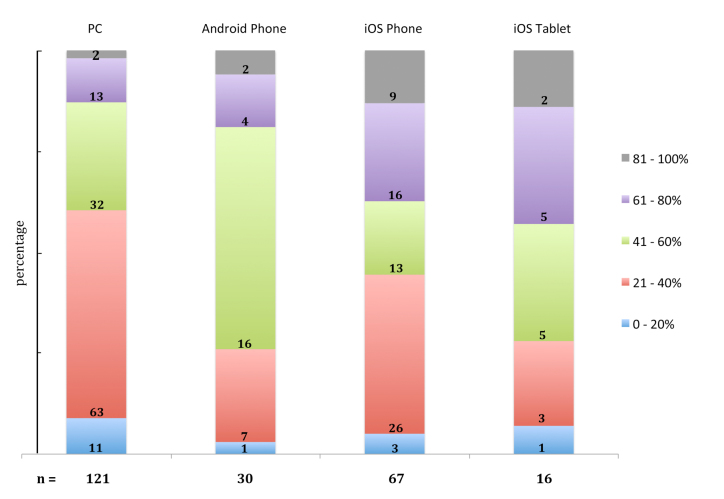
Activity of Internet based learning performance related to the internet devices PC, Android Phone, iOS Phone and iOS Tablet, respectively. The number of students utilizing a specific device is indicated by “n”. The application of each device employed for learning activities varies between students. This variation causes an alignment in 20 percent steps (see coloured boxes at the right). Thus, the absolute frequency is displayed in “grey” (81 – 100%), the lowest frequency in “blue” (0 – 20%). The actual number of students referring to the individual frequencies is given for each Internet device. This number directly relates to the variable size of coloured boxes and can be transferred in percentage (ordinate).

Internet-based learning activities are linked to specific e-learning devices as detailed in Figure 1. Analyzing the utilization of computers (PCs) for e-learning it became apparent that the range of 21 – 40 % of internet-based learning activity of students is linked to half (52%) of computers available. Interestingly, as the internet learning activity increases (range 41 – 100 %), the preference to utilize computers clearly declines. At the highest level of e-learning efforts (81 – 100 %) less students made use of computers (PCs). Noteworthy differences were observed to use tablet PCs and smartphones for e-learning. The documentation for tablet PCs (iOS Tablets) reveals that the e-learning activity (range from 21 to 80 % activity) is related to either 77 % tablet PCs equipped with iOS or to 100 % tablets equipped with Android OS. Remarkably, those students conducting almost entirely e-learning activities (81 – 100 %) during their clinical education preferred iOS tablets and iOS phones (27 %), only 8 % utilized PCs and Android phones. However, tablet PCs carrying iOS are most popular for internet-based learning activities. A break down of data compiled for smartphones (Android phone, iOS phone) displays some distinct differences when compared to the use of computers or iOS tablets. Again, slightly more than one-third of students performing e-learning activities (21 - 40 %) is using an iOS smartphone (39%). Smartphones equipped with Android OS are absolutely favored for e-learning. These devices are employed to 91 % by students performing e-learning activities in the range of 21 to 80 %. Our data do not allow to transfer percentages of users directly in an absolute number of probands as individual students utilize more than 1 e-device for e-learning activities. This is inferred from the number of students (n=141) participating in the survey compared with the total of e-learning devices students (n=234) employ (see Table 1).

Utilization of e-learning programs is allocated to specific hardware-types. Since e-learning hardware devices are without exception highly sophisticated technical allround tools one would not necessarily expect a preference of any type of device for a specific e-learning program. From 121 students using computers (PCs) for their e-learning activity, 37 % (n=5) took advantage of learning programs. A different outcome holds for tablet PCs. From students owning Tablet PCs equipped with iOS only 54% (n=9) employed e-learning programs. Marginal acceptance was found for tablets PCs furnished with Android OS, and only 1 proband used an assigned learning program. Data for smartphones are divergent from computers and tablets. Students employing iOS-smartphones about half of them (55%, n=37) practice appropriate e-learning programs. From Android smartphone users only 14 % (n=4) took advantage of e-learning programs. To avoid an overinterpretation of the data presented here we suggest that of all students questioned only one-third regulary uses e-learning programs, regardless of the different hard ware devices and OSs employed.

## Discussion

### Summary

The data collected from 141 dental students reveal that all utilize at least 1 internet hardware device such as PC, smart phone or tablet PC, and they have all unlimited access to the internet. The overwhelming part of probands (90%) employ laptops for e-learning, while only three-quarter of mobile device owners use this hard-ware for e-learning activities. The more intense students are engaged in e-learning, the more they use smart phones and tablets PCs. Students performing almost exclusively e-learning favour highly sophisticated e-learning hardware like smart phones and tablet PCs equipped either with Android OS or with iOS. e-Learning programs are less popular. Most students are operating PCs but only 30 % utilize e-learning programs. The acceptance for e-learning software increases to 50 % as high-class devices (iOS equipped tablet PCs, Android Phones) are employed.

This outcome of our survey conducted with 141 dental students during their clinical education describes a current state of hard and software employed for e-learning activities. Although the whole issue is subject to rapid developments, a comparison with previous data [6] discloses a clearly increased application of e-learning techniques. This holds both for the access to the Internet and to the availability of hardware devices. The data gathered here implicate that dental students overwhelmingly favour PCs (laptops) for e-learning activities, which we relate primarily to the formidable sales figures of this device. However, a trend is apparent that the availability of more sophisticated hardware, such as smart phones and tablet PCs equipped with efficient OSs, promotes the attractiveness of e-learning. In fact, students highly engaged in e-learning (81 - 100 %) favour for their efforts high-class devices. In general, this view is supported by our survey as the relative use of iOS equipped tablet PCs is increasingly employed for e-learning activities (range from 41 - 100%). A somewhat different result was obtained with respect to smartphones. About half e-learning activities of student relates to iOS smartphones. Smartphones equipped with Android OS often exhibit enlarged screens as compared to iOS phones, and this fact seems to attract students performing an intense internet-based learning activity. Again, the reliability of this result should be judged cautiously as the phone-purchasing behavior of students may change rapidly with new applications not confined to e-learning matters. Despite these conjectures we assume that high tech preconditions are most relevant to improve clinical education of dental students.

Utilization of e-learning programs was found mostly accepted by students owning iOS tablet PCs and iOS smartphones, respectively. This finding contrasts data for tablet PCs and smartphones equipped with Android OSs, exhibiting a moderate use (about 20%) of learning programs. Unfortunately, the responses obtained for tablet PCs is rather low impairing a reliable interpretation. Rather convincing appear the results regarding computers (desktop, laptop). About one third out of 141 students employs learning programs installed on computers. This supports the notion and let us suggest that affordable prices and an easy access to e-learning programs is eligible to promote their widespread dissemination. Regardless of the preferences dental students exhibit for e-learning devices, the trend continuous to an increased engagement in e-learning activities linked to contemporary techniques [14].

### Conclusions

Our survey confirms recent suggestions that dental students increasingly accept e-learning offers. The essential preconditions to perform these studies are excellent, since all students have access to computers (desktop, laptop) and mobile e-learning devices (tablet PC, smartphone), respectively. A trend to tablet PCs and smartphones equipped with high performance operation systems appears evident. While the survey participants are overwhelmingly engaged in e-learning activities, only one-third to half of students deploys e-learning programs. We conclude that e-learning activities of dental students meanwhile represent an intergral part of clinical training at the University Mainz.

## Abbreviations

PC: personal computers
OS: operation system
iOS: iPhone operating system
